# Listening to yourself is special: Evidence from global speech rate tracking

**DOI:** 10.1371/journal.pone.0203571

**Published:** 2018-09-05

**Authors:** Merel Maslowski, Antje S. Meyer, Hans Rutger Bosker

**Affiliations:** 1 Max Planck Institute for Psycholinguistics, Nijmegen, The Netherlands; 2 International Max Planck Research School for Language Sciences, Nijmegen, The Netherlands; 3 Donders Institute for Brain, Cognition and Behaviour, Radboud University, Nijmegen, The Netherlands; University of Amsterdam, NETHERLANDS

## Abstract

Listeners are known to use adjacent contextual speech rate in processing temporally ambiguous speech sounds. For instance, an ambiguous vowel between short /α/ and long /a:/ in Dutch sounds relatively long (i.e., as /a:/) embedded in a fast precursor sentence, but short in a slow sentence. Besides the local speech rate, listeners also track talker-specific global speech rates. However, it is yet unclear whether other talkers’ global rates are encoded with reference to a listener’s self-produced rate. Three experiments addressed this question. In Experiment 1, one group of participants was instructed to speak fast, whereas another group had to speak slowly. The groups were compared on their perception of ambiguous /α/-/a:/ vowels embedded in neutral rate speech from another talker. In Experiment 2, the same participants listened to playback of their own speech and again evaluated target vowels in neutral rate speech. Neither of these experiments provided support for the involvement of self-produced speech in perception of another talker’s speech rate. Experiment 3 repeated Experiment 2 but with a new participant sample that was unfamiliar with the participants from Experiment 2. This experiment revealed fewer /a:/ responses in neutral speech in the group also listening to a fast rate, suggesting that neutral speech sounds slow in the presence of a fast talker and vice versa. Taken together, the findings show that self-produced speech is processed differently from speech produced by others. They carry implications for our understanding of rate-dependent speech perception in dialogue settings, suggesting that both perceptual and cognitive mechanisms are involved.

## Introduction

Self takes a special role in the processing of cognitive and perceptual information. For instance, one’s own face is recognized faster and more accurately than other familiar and unfamiliar faces [1]. Also, self-relevant stimuli, such as self-owned or self-associated items, attract more attention compared to stimuli associated with others [2–6]. Not only self-relevant, but also self-produced items aid processing: Stroke recognition in hand-writing is facilitated when strokes are self-produced [7, 8]. Additionally, words were remembered better when participants read them aloud themselves during encoding than when they heard them read by others [9]. This advantage of self-produced words remains even when participants’ own voices are recorded earlier and played back to them at test.

However, whether and how self-produced items influence perception of other-produced items is less well studied. The most common situation in which humans constantly switch between experiencing self-produced and other-produced input is dialogue. In dialogue, interlocutors easily alternate between speaking and listening, with turn gaps being remarkably short (∼200 ms) [10]. Given that one’s own speech often constitutes the context for an interlocutor’s utterance, self-produced speech may affect the perception of the speech from another talker. The present study investigated how our own speech production, and specifically self-produced speech rate, affects how we process temporal cues in speech from another talker. The study replicates prior work on speech rate effects in global speech contexts and provides new empirical evidence that self-produced speech is processed differently from speech produced by others. As such, it contributes to our understanding of the representation of one’s own voice in dialogue.

Temporal features of speech vary considerably with speech context. One reason for this is that acoustic cues map differently onto phonemic categories at different speech rates. Listeners must therefore normalize for contextual speech rate in order to interpret temporally ambiguous speech sounds [11–18]. That is, temporal cues in the ongoing acoustic signal are perceived relative to the surrounding speech rate, such that the signal can be identified as a meaningful linguistic object (a segment, syllable, or word). Therefore, perception of speech sounds that mainly differ temporally, such as short and long vowels (e.g., German /a/ and /a:/) [19] or consonants (e.g., English /b/ and /w/) [20] can shift from one phoneme to another based on contextual speech rate. For instance, an ambiguous vowel midway between Dutch /α/ and /a:/ is biased towards short /α/ in a slow speech context [21], as the adjacent speech rate makes the vowel sound relatively short (i.e., as /α/ in “stad” [stαt] *city*). Similarly, the same ambiguous vowel is biased towards long /a:/ in fast speech contexts, where it sounds relatively long (i.e., as /a:/ in “staat” [sta:t] *state*). This phenomenon is referred to as rate normalization and it is the process that we investigate here in relation to self-produced speech.

Temporal cues can be affected both by the local surrounding sentence context and more global speech contexts. Most studies so far have focused on local context effects; that is, effects of an adjacent sentence. Such local rate-dependent context effects have been argued to involve general auditory mechanisms. For instance, they have been shown to occur independently of talker voice changes [22, 23], with a fast speech context from talker A influencing subsequent perception of a target by talker B. Moreover, the speech-like nature of the context seems to be inconsequential; both speech and non-speech induce local rate effects [11, 24, 25]. Context effects have furthermore been shown to hold even for 2–4 months old infants [26] and non-human species [27]. Lastly, effects of adjacent rate contexts are unaffected by attentional modulation, which supports the involvement of early perceptual processes [28].

**Fig 1:**
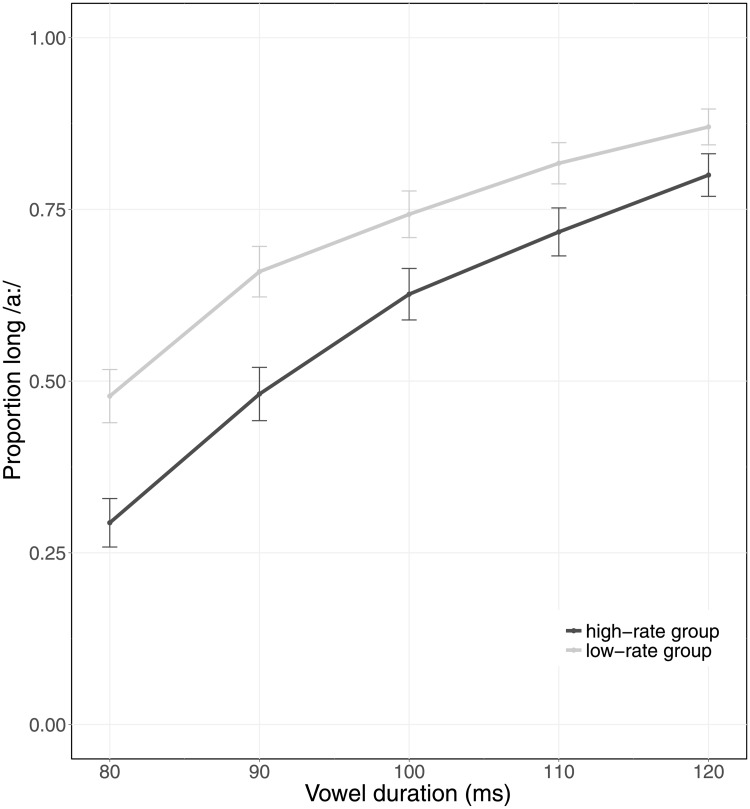
Adapted reprint of only the neutral rate average categorization data of Maslowski et al.’s [31] Experiment 2. The X-axis indicates Vowel Duration (80–120 ms). Colors indicate Group, with the high-rate group shown in dark grey and the low-rate group shown in light grey. Error bars represent the 95% confidence intervals. Adapted from “How the tracking of habitual rate influences speech perception,” by M. Maslowski, A. S. Meyer, and H. R. Bosker, 2018, Journal of Experimental Psychology: Learning, Memory, and Cognition.

However, language users are also sensitive to talker-specific variation [29, 30]. More global effects of speech rate (induced by cues from non-adjacent larger speech contexts and multiple talkers) seem to be sensitive to such higher-level influences such as talker voice. Maslowski, Meyer, and Bosker [31] investigated whether one talker A’s global rate is perceived relative to another talker B’s speech rate. In their experiments, two groups of participants listened to sentences spoken by a male and a female talker. In one experiment, examining effects of talker-specific global speech rate, a high-rate group listened to one talker A speaking at a high speech rate and another talker B speaking at a ‘neutral’ speech rate. A second group, the low-rate group, listened to the same neutral speech rate (talker B), but to talker A speaking at a low speech rate. On each trial, participants categorized a word with a temporally ambiguous vowel between Dutch /α/ and /a:/ (e.g., /takjǝ, ta:kjǝ/, “twig”/“task”) that was embedded in a trial sentence (e.g., *Toen Luuk mompelend iets tegen Lotte vertelde, hoorde Lotte “het takje/taakje” gezegd worden*, “When Luuk muttered something to Lotte, Lotte heard “the twig/task” being said”). The two participant groups were then compared on their perception of these vowels in sentences from talker B speaking at a neutral rate. That is, whilst the local rate cues in talker B’s speech were identical for both groups, the global context (fast/slow speech from other talker) in which talker B was heard differed between groups. The authors observed an effect of global speech rate; in the high-rate group listening to a fast talker A, they observed significantly fewer /a:/ responses in neutral talker B’s speech, compared to the low-rate group with a slow talker A (reproduced in Fig 1). This suggests that B sounded slow when A was faster, but fast when A was slower.

Interestingly, the effect disappeared in another experiment, where each talker spoke at two speech rates in separate trials. That is, the high-rate group heard fast and neutral rate speech from talker A as well as from talker B. Conversely, the low-rate group heard slow and neutral speech from talker A and B. As a consequence, there was large intra-talker variability in speech rate, in contrast to the previous experiment where the intra-talker speech rates were held constant. However, the average rate across the two talkers in the two groups was identical in both experiments. The results of this experiment showed no difference between the two groups in the proportion of /a:/ responses in neutral speech. The authors interpreted their results from the two experiments together as listeners tracking talker-specific global rates rather than a talker-independent average rate. That is, with sufficient intra-talker regularity, cues to global speech rate are used in perception of another talker, with the global rates from different talkers being perceived relative to each other.

A question arising from these experiments on local and global rate effects is how talkers’ self-produced speech rates are represented in comparison to other talkers’ rates. When producing speech, talkers also hear themselves speaking, with their own speech typically comprising the context for their interlocutor’s speech. Therefore, a listener’s self-produced speech rate may modulate perception of the global rate of another talker. Alternatively, global rate tracking may involve operations that factor out self-produced rate, as self-produced speech is not necessarily informative in disambiguation of speech from other talkers.

We already know that in local contexts, speech rate effects can be induced by self-produced speech. In a recent study by Bosker [22], participants were instructed to read out sentences at two pre-specified rates (fast and slow). Immediately after participants had produced a sentence themselves, they heard an ambiguous target word (/α/ vs. /a:/) produced by someone else. Bosker observed a difference in perception of target words between the condition in which participants were instructed to speak fast and the condition in which they had to speak slowly; more long /a:/ responses were observed when participants spoke fast. This suggests that self-produced speech can affect perception of speech produced by others. No speech rate effect was observed in another experiment testing effects of fast and slow covert speech (produced silently without articulation). That is, the effect of overt self-produced speech seems to be a consequence of self-monitoring of the external signal.

Noteworthy, Bosker’s [22] study also included an experiment in which the recordings from the previous experiment on self-production were played back to the same participants (passive listening to one’s own voice). Here, the difference between the fast and the slow condition was somewhat larger than in the first experiment. Bosker speculated that the smaller effect of self-production in the first experiment (relative to passive listening in the other experiment) may be due to speaking-induced suppression (SIS). SIS involves a reduction in the neural response to self-produced speech in auditory cortex (as compared to the neural response to perception without production, i.e., playback of one’s own voice) [32, 33]. It may be that SIS attenuates the processing of self-produced speech rate during production compared with passively listening to self-produced speech.

Building on Bosker [22], the present study investigates how self-produced speech may affect *global* speech rate perception, using the design and materials from Maslowski et al. [31]. Experiment 1 tested whether self-produced speech rate plays a part in perception of another talker’s global rate. The experiment featured equal proportions of production and perception trials. One group of participants (high-rate group) was instructed to produce sentences at a fast speech rate (production trials) and to categorize words in ‘neutral’ rate sentences from another talker (perception trials). Another group (low-rate group) spoke at a slow rate (production trials) and listened to the same neutral speech from the other talker (perception trials). The two groups differed only in the rate at which they produced sentences in production trials. The production trials were mixed with perception trials from the other talker, which contained an ambiguous Dutch /α/-/a:/ vowel embedded in minimal pairs that were only distinguishable by this vowel.

If listeners perceive the global speech rate of another talker relative to their self-produced speech rate, categorization responses should differ between the high-rate group and the low-rate group. The high-rate group should then report hearing fewer long /a:/ vowels than the low-rate group, because the neutral talker sounds relatively slow. Such a finding would mirror that of the global rate experiment in Maslowski et al. [31]. However, if listeners base their perception of global speech rate only on other talkers’ speech and do not rely on their own productions, no group difference should be observed in /α/–/a:/ word categorization in neutral speech in this experiment.

To preview the findings, the results of Experiment 1 showed no group difference. This result could be a consequence of the production task itself, corresponding to the attenuated self-produced rate effects (relative to passively listening to oneself) in Bosker [22]. Bosker speculated that the smaller effect of self-produced speech rate could be a consequence of SIS. If the null result of our Experiment 1 is indeed due to SIS, an effect of self-produced rate may emerge when no production task is involved. That is, listening to playback of self-produced speech may modulate perception of another talker.

Alternatively, self-awareness may lead listeners to factor out their own speech, whether they are listening to themselves during production or listening to themselves passively. Listeners would recognize their own voice when listening to playback of their own speech. Because self-produced speech rate is typically uninformative for the perception of others, listeners may ignore their own productions when interpreting speech from another talker. This account would predict no effect of passively listening to self-produced speech rates (i.e., no group effect).

To distinguish between these two accounts, in Experiment 2, the participants from the previous experiment were invited back to listen to their own speech recorded in Experiment 1. The experiment was identical to the first experiment, except now production trials were replaced with playback of the recordings of the same self-produced trials. If listening to oneself passively is different from listening to oneself whilst speaking (for instance as a result of SIS), the results of this experiment should deviate from those of Experiment 1 (i.e., a group difference, with the high-rate group reporting fewer long /a:/ responses). However, if it is not the input itself, but rather the fact that the input was self-produced that led to the lack of an effect in the previous experiment (because of self-awareness), the results of this experiment should parallel those of Experiment 1. To preview findings once more, no effect of passively listening to self-produced speech was found in Experiment 2 (i.e., no difference between the two groups). This may suggest that the null result in Experiment 1 was not a consequence of SIS, but rather self-awareness.

An alternative interpretation of the null results found in Experiment 1 and 2 is that the results stem from variability within the fast and slow rates produced by the previous participants. Maslowski et al. [31] found that when intra-talker variability is increased, global rate effects disappear. Similarly, because the speech produced in fast and slow production trials in Experiment 1 naturally included some intra-talker variability (within limits), this may have eliminated the global rate effects of the previous experiments (i.e., compared to the highly controlled and artificially compressed and expanded fast and slow materials in Maslowski et al.’s Experiment 2).

If the null results observed in Experiments 1 and 2 were due to intra-talker variability within the fast and slow rates, no global rate effect should emerge when Experiment 2 is repeated with a different participant sample, who are unfamiliar with the voices from the participants from before. However, if the results in the preceding experiments were indeed due to self-awareness, this would predict an effect of global speech rate (as found in Maslowski et al. [31], with the same neutral rate materials) when presented to different participants. Therefore, in Experiment 3, Experiment 2 was repeated with a new participant sample, who did not know the participants from before. As such, each participant heard one talker speaking at a neutral rate in perception trials and passively listened to (fast or slow) production trials from one of the participants from Experiment 1. After each neutral rate trial, participants again evaluated an /α/-/a:/ vowel in a target word. We predicted that the results of the experiment would replicate the findings in Maslowski et al.: Listening to a fast talker A and a neutral talker B, should make talker B sound relatively slow, whereas listening to a slow talker A should make talker B sound relatively fast.

## Experiment 1: Self-production

Experiment 1 addressed the question whether self-produced speech rate affects perception of other talkers in global speech contexts. On the one hand, this may not be the case, as tracking self-produced rate may not necessarily be useful for comprehension of other talkers. On the other hand, self-produced speech may affect perception of others in global speech contexts in the same way as the global speech rate of one talker A influences perception of the speech rate of another talker B [31]. Moreover, effects of self-produced speech have previously been found in local contexts [22], with one’s own voice affecting subsequent perception of an immediately following target produced by another talker.

### Methods

#### Participants

A sample of native Dutch female participants (*N* = 41, *M*_*age*_ = 23, range = 19–33) with no hearing, visual, or reading deficits were recruited from the Max Planck Institute participant pool. All gave their informed consent to participation, as approved by the Ethics Committee of the Social Sciences department of Radboud University (project code: ECSW2014-1003-196). A priori, it was decided to exclude participants with a proportion of /a:/ responses of < 0.1 or > 0.9, applying the same criterion as in our prior study [31], from which the stimulus set was adopted. Data from 9 participants were excluded, either because they performed outside the aforementioned range (*n* = 7) or because of non-compliance (e.g, frequently talking to themselves during perception trials; *n* = 2).

#### Design and materials

Experimental materials consisted of the ‘neutral rate’ materials used in Maslowski et al. [31]. These materials comprised eight 24-syllable sentences with one of two Dutch /α/-/a:/ minimal pairs: *takje*/*taakje* (/takjǝ, ta:kjǝ/, “twig”/“task”) and *stad*/*staat* (/stαt, sta:t/, “city”/“state”) (e.g., *Terwijl Niels rustig zijn tijdschrift stond te lezen, hebben de heren eens “stad/staat” tegen hem gebruld*, “Whilst Niels was peacefully reading his magazine, the gentlemen roared “city/state” to him once”). None of these sentences contained other instances of the vowels /α, a:/, nor did they bias either member of a minimal pair semantically. The sentences were recorded by a native Dutch male and a female talker. All speech up to a target vowel was set to the mean duration of that interval across the two talkers, using the PSOLA algorithm as implemented in Praat [34]. Similarly, all speech after vowel offset was matched across the two talkers.

In Dutch, the /α, a:/ vowel contrast is acoustically differentiated both temporally and spectrally, with /α/ being short with a relatively low F2 and /a:/ being long with a high F2 [35]. To construct vowel duration continua, one clear long vowel /a:/ from each talker was extracted. The vowel duration continua were created by linear compression using PSOLA and ranged from 80 to 120 ms (in five steps of 10 ms). To make the vowels spectrally ambiguous, the F1s and F2s from both talkers were computed and set to a fixed ambiguous value using Burg’s LPC algorithm in Praat (male talker: F1 of 764 Hz and F2 of 1261 Hz; female talker: F1 of 728 Hz and F2 of 1327 Hz). For each sentence, target vowels were then concatenated with the intervals before and after the target vowel, resulting in a stimulus set of 80 unique stimuli (8 context phrases × 5 vowel durations × 2 talkers). For more details on stimulus construction, see Maslowski et al. [31].

#### Procedure

The experimental procedure consisted of production trials and perception trials. Participants were randomly divided into two groups (both *n* = 16), who were both presented with an equal number of perception trials and production trials. The perception trials were identical across groups. A perception trial involved listening to ‘neutral rate’ speech from one of the two talkers (male or female), after which a button press response was required to indicate which member of a minimal pair the participant had heard in the sentence. Talkers were counterbalanced across participants.

Each perception trial started with a fixation cross (for 330 ms) that was always replaced by a stimulus sentence shown on the screen in black on a white background at auditory onset. The target word in the stimulus sentence was replaced by a question mark (e.g., *Terwijl Niels rustig zijn tijdschrift stond te lezen, hebben de heren eens [?] tegen hem gebruld*). At sentence offset, this screen was replaced by a screen showing two response options (e.g., *stad* and *staat*). For the word shown on the left side of the screen, participants pressed “1” and for the word on the right they pressed “0”, with the position of the response options on the screen being counterbalanced across participants. Participants had 4 seconds to respond by button press, before a missing response was recorded.

Crucially, the two groups differed on production trials, which were randomly intermixed with perception trials. A production trial involved reading out a sentence at a pre-specified speech rate. Participants in the high-rate group had to produce speech at a fast speech rate, whereas participants in the low-rate group produced speech at a slow articulation rate. These pre-specified speech rates were based on the durations of fast trials (1/1.6 = 0.625 × the durations of neutral trials) and slow trials (1.6 × the durations of neutral rate trials) in the experiments in Maslowski et al. [31]. Participants were explicitly instructed to speak without pausing between words. The sentences participants were instructed to read out in the production trials were the same as those in the perception trials, except that the target words *takje*/*taakje* and *stad*/*staat* in the production items were substituted by *tukje* (/tYkjǝ/, “nap”) and *stoet* (/stut/, “procession”), to prevent participants’ own /α, a:/ vowels from affecting the perception of /α, a:/ in the perception trials.

Production trials were cued by showing the sentences in red. After 1200 ms, the sentence turned green, to prompt the participant to start speaking. During production trials, recordings were made of the participant’s speech. The experimenter could hear the participant through headphones throughout the experiment. After 0.625 (high-rate group) or 1.6 (low-rate group) times the durations of the neutral rate stimuli, a beep was played to the experimenter (inaudible to the participant). When the participant had finished producing the sentence, the experimenter pressed a button to give feedback on the participant’s rate as indicated by the beep (“Please try to speak somewhat faster”/“Please try to speak somewhat slower”/“Well done!”).

Prior to the experiment, participants completed two separate practice blocks, one for each modality. In the listening practice, each of the eight different sentences with various instances of the vowel continua endpoints were presented once. In the production practice, all eight sentences (with the substituted target words) were presented once in a practice block, but this block was repeated until the participant successfully produced them at the pre-specified rate.

Stimulus presentation was controlled by Presentation software (v16.5; Neurobehavioral Systems, Albany, CA, USA). Stimuli were presented in five blocks of 80 trials. Each block consisted of a random mix of all unique auditory stimuli of one talker (perception trials: *n* = 40) and five instances of each individual production item (production trials: *n* = 40), resulting in 200 perception trials and 200 production trials in total. One session lasted for a duration of approximately 55 minutes in the high-rate group, and 70 minutes in the low-rate group. After the experiment, participants indicated that they could clearly hear their own voice, despite wearing headphones.

### Results

#### Production

**Fig 2:**
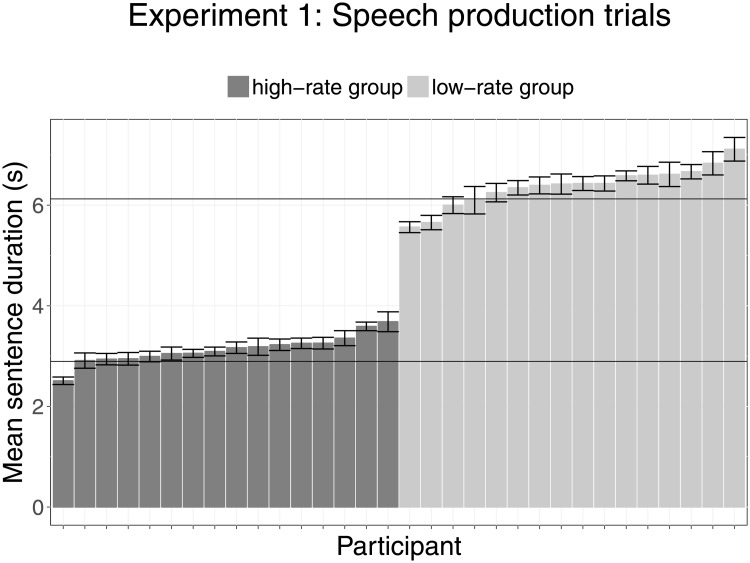
Mean sentence durations of speech production trials of Experiment 1 (self-production). On the X-axis, sentence durations are given for each participant in ordered sequence. Colors indicate Group, with the high-rate group shown in dark grey and the low-rate group in light grey. The horizontal lines indicate the intended sentence duration for the high-rate group (bottom) and the low-rate group (top). Error bars represent the 95% confidence intervals.

The participants’ sentence durations on production trials were analyzed as a proxy for speech rate. Production trials were disregarded from analysis when they contained word errors, coughs, or pauses of > 500 ms in the low-rate group and > 200 ms in the high-rate group. In total, 18.6% of production trials were excluded in the low-rate group and 14.6% of trials in the high-rate group, mainly due to pauses and word errors in the low-rate group and the high-rate group, respectively. Fig 2 illustrates the mean duration of production trials for each participant in the high-rate and low-rate groups. This figure shows a relatively small difference in speech rate within the two groups and a clear separation between groups, as confirmed by a paired-samples t-test (*t*(30) = 18.503, *p* < 0.001, *d* = 12.53) comparing the mean durations of production trials between the high-rate group (*M* = 3122 ms, *SD* = 347 ms) and the low-rate group (*M* = 6344 ms, *SD* = 604 ms). This verifies that participants complied with our instructions.

#### Perception

**Fig 3:**
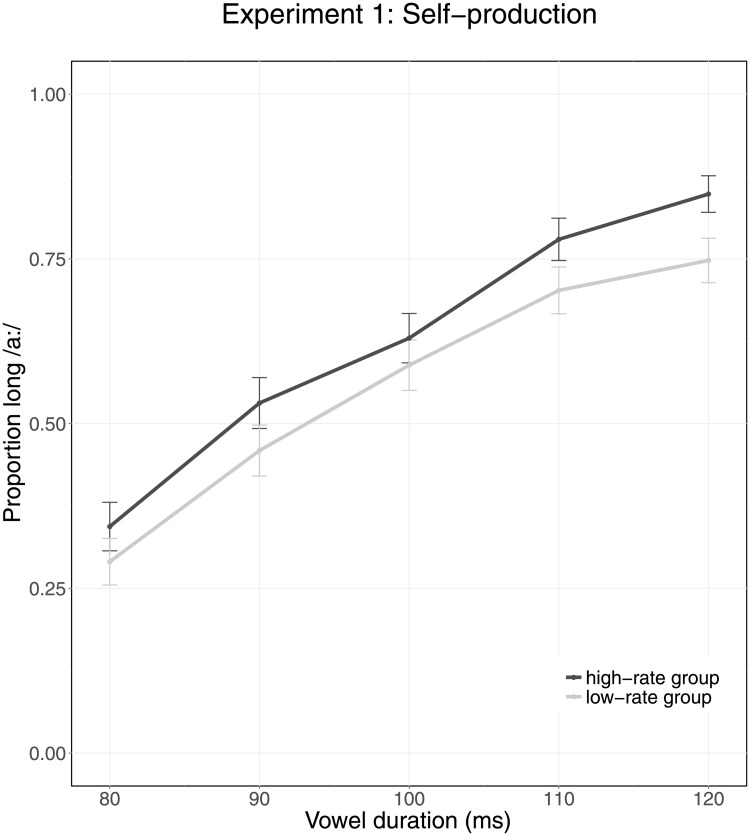
Average categorization data of Experiment 1 (self-production). The X-axis indicates Vowel Duration (80–120 ms). Colors indicate Group, with the high-rate group shown in dark grey and the low-rate group shown in light grey. Error bars represent the 95% confidence intervals.

Fig 3 shows the categorization data on perception trials (proportion /a:/ responses) in Experiment 1. Participants reported a lower proportion of /a:/ when target vowels were at the shorter end of the duration continuum and a higher proportion when they were at the longer end of the continuum. Although Fig 3 seems to suggest that the two groups may differ slightly in their perception of vowels embedded in neutral speech in the opposite direction of our prediction, the following statistical analysis showed otherwise.

The binomial responses to the perception trials (0.03% missing responses excluded) were quantified statistically with a Generalized Linear Mixed Model (GLMM) with a logistic linking function from the lme4 package [36] in R [37]. This GLMM tested whether there was a difference between the two groups in perception of ambiguous vowels embedded in neutral speech. Unless otherwise stated, the same full model was used in all three experiments and included the predictors Group (categorical; intercept is high-rate), Vowel Duration (continuous; centered and divided by one standard deviation), Block (continuous; centered and divided by one standard deviation), and Talker (categorical; sum-to-zero coded). The included interactions between fixed factors were between Group and Vowel Duration, Group and Block, and Vowel Duration and Block. Random intercepts were included for Participants and Items with random slopes for all predictors by both random effects, except for the control variable Talker. Because the full model failed to reach convergence for the current experiment, the random slopes for Vowel Duration and Block were dropped for both random effects.

Vowel Duration significantly affected the proportion of /a:/ responses (*β* = 0.974, *z* = 20.649, *p* < 0.001), with vowels of longer durations more often being reported as /a:/ than shorter vowels. The predictor Group did not reach significance (*β* = −0.374, *z* = −1.316, *p* = 0.188), providing no evidence for an effect of self-produced speech rate on the perception of another talker. The dependent variable proportion of /a:/ responses was also significantly affected by Block (*β* = −0.156, *z* = −3.614, *p* < 0.001), indicating that participants perceived decreasingly fewer /a:/ vowels as the experiment went on. The control variable Talker reached significance (*β* = 0.889, *z* = 2.795, *p* = 0.005), with an overall significantly higher proportion of /a:/ responses for the female talker. There were no significant interactions between fixed factors (Groups and Vowel Duration: *β* = −0.036, *z* = −0.542, *p* = 0.586; Groups and Block: *β* = −0.023, *z* = −0.369, *p* = 0.712; Vowel Duration and Block: *β* = 0.006, *z* = 0.194, *p* = 0.846).

The fact that no effect of self-production was observed (i.e., no group effect) could be because effects of one’s self-produced speech rate are more short-lived than effects of others’ global rates. Therefore, a more fine-grained analysis was performed on a subset of the data, consisting of only the perception trials directly following a production trial (*n* = 3258, 51.0%). However, no qualitative differences were observed compared to the results of the data in the full set.

The results of this experiment provide no evidence that target word perception in the perception trials was sensitive to participants’ self-produced speech rates in production trials, suggesting that self-produced speech does not affect perception of another talker’s global speech rate. This is in contrast to the results in Maslowski et al. [31]. In their Experiment 2, participants evaluated the same neutral rate trials, but (instead of the present production trials) listened to another talker producing speech at a consistently fast/slow speech rate. They found an effect of global speech rate on the perception of another talker. However, replacing their fast and slow perception trials with the fast and slow production trials here seemed to remove the effect. This suggests that listening to oneself whilst talking has a different effect on perception than passively listening to another talker.

Additionally, the results of Experiment 1 differ from Bosker [22], who compared participants’ vowel categorization immediately after having produced either fast or slow speech. In this very local self-produced speech context, Bosker observed a difference between participants’ perception of target words. Together, these studies suggest that self-produced speech induces a bias in local (adjacent) speech contexts, but not in global (distant) speech contexts.

## Experiment 2: Playback self-production

Experiment 2 tested whether the lack of an effect of self-produced speech in Experiment 1 could be related to the production task itself. Auditory input from self-produced speech has been found to lead to reduced responses in auditory cortex (i.e., speaking-induced suppression; SIS), which may in turn have reduced the magnitude of a potential effect of self-produced global speech rate. As such, SIS could be argued to account for the lack of a shift in phonetic categorization in Experiment 1. Therefore, Experiment 2 repeated the experiment without a speech production task, by having the same participants listen to playback of their own speech (produced in Experiment 1).

### Methods

#### Participants

The same sample of participants as in Experiment 1 was invited back to participate in Experiment 2. Out of the 32 participants from Experiment 1 who were included in the analyses, 22 returned for Experiment 2 (high-rate group: *n* = 10; low-rate group: *n* = 12). Group sizes were mildly unbalanced.

#### Design and materials

The same materials were used as in Experiment 1. This included all production trials (including word errors, coughs, and pauses) and all perception trials.

#### Procedure

The procedure of Experiment 2 was identical to Experiment 1, except now participants listened to playback of their own 200 sentence recordings from the previous experiment, instead of producing speech. As before, participants were only prompted to respond after neutral rate trials from the other talker. After playback of a self-produced speech trial, the next trial was presented directly. Participants were aware that half of the stimuli were self-produced. They listened to experimental stimuli and self-produced stimuli in the exact same order as presented and recorded in Experiment 1.

### Results

**Fig 4:**
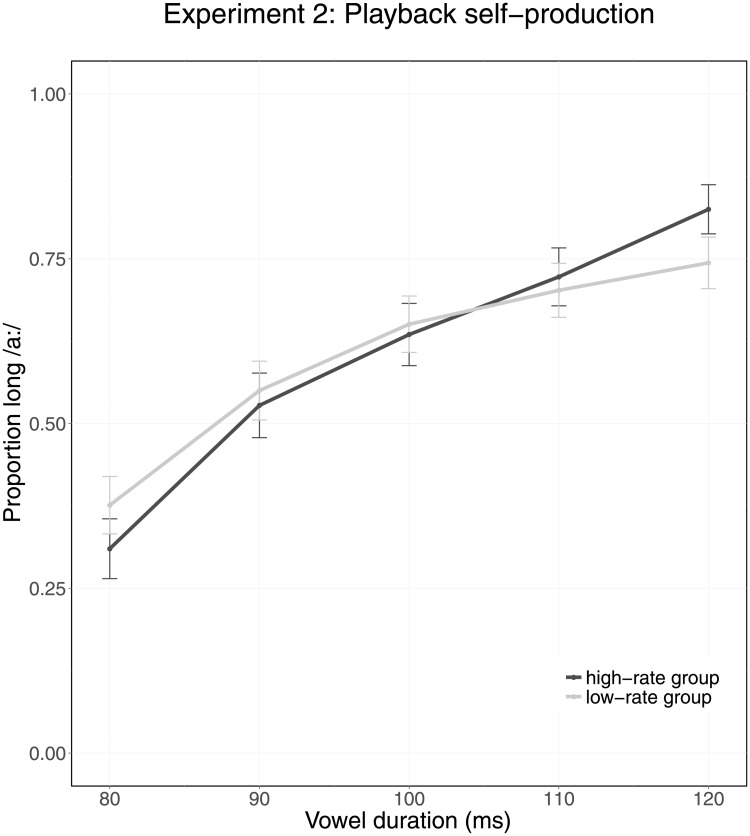
Average categorization data of Experiment 2 (playback self-production). The X-axis indicates Vowel Duration (80–120 ms). Colors indicate Group, with the high-rate group shown in dark grey and the low-rate group shown in light grey. Error bars represent the 95% confidence intervals.

Fig 4 summarizes the categorization data of Experiment 2. The figure indicates that participants reported hearing a higher proportion of /a:/ for targets with longer vowel durations. The overlap of the two lines suggests that there is no difference between the categorization data of the two groups.

The categorization data of Experiment 2 were tested with the full GLMM as described in Experiment 1, except that the random slopes for Block were dropped due to convergence issues. Vowel Duration significantly affected the proportion of /a:/ responses (*β* = 1.261, *z* = 4.699, *p* < 0.001), with participants more often reporting hearing /a:/ for longer vowel durations. Group had no significant influence on /a:/ categorization (*β* = 0.096, *z* = 0.199, *p* = 0.843), suggesting that the likelihood of hearing /a:/ in neutral speech was the same for both groups. Block did not reach significance (*β* = −0.043, *z* = −0.745, *p* = 0.456), showing that performance did not change over time. However, the interaction between Vowel Duration and Block was significant (*β* = 0.104, *z* = 2.511, *p* = 0.012), with the difference in /a:/ categorization between the two endpoints of the duration continuum being larger in later blocks than earlier ones. Talker also significantly affected categorization (*β* = 1.229, *z* = 2.340, *p* = 0.019), with a higher proportion of /a:/ for the female talker. Finally, the interactions between Group and Vowel Duration (*β* = −0.175, *z* = −0.555, *p* = 0.579) and Group and Block (*β* = 0.060, *z* = 0.755, *p* = 0.450) were not significant.

Experiment 2 tested whether listening to playback of self-produced fast or slow speech induces variation in speech perception of another talker (speaking at a neutral speech rate). No effect of listening to one’s own speech was found on the perception of another talker. This suggests that the lack of an effect of self-produced speech in Experiment 1 was not due to speaking and listening at the same time. Moreover, the result contrasts with Maslowski et al. [31], who found effects of talker-specific global speech rate. Therefore, this finding suggests that listening to oneself is intrinsically different from listening to other talkers.

## Experiment 3: Unfamiliar listeners

Experiment 3 aimed to evaluate whether the null results from the previous experiments were related to participants hearing themselves (rather than another talker). Therefore, Experiment 2 was repeated but with a new sample of participants, who listened to the fast of slow sentence recordings made in Experiment 1 and evaluated neutral rate sentences.

### Methods

#### Participants

Native Dutch female participants (*N* = 40, *M*_*age*_ = 22, range = 19–27) were recruited and divided into a high-rate group (*n* = 16) and a low-rate group (*n* = 16). All gave their consent to participation. Data from eight participants were excluded, because their responses were outside the performance criterion described in Experiment 1, resulting in two pseudo-random groups of 16 participants each.

#### Design and materials

The same materials were used as in Experiment 2, including the self-produced trials from the participants from Experiment 1.

#### Procedure

The procedure was identical to that of Experiment 2, with the only difference between Experiment 2 and 3 being that, in Experiment 3, participants listened to speech from (to them) unfamiliar talkers.

### Results

**Fig 5:**
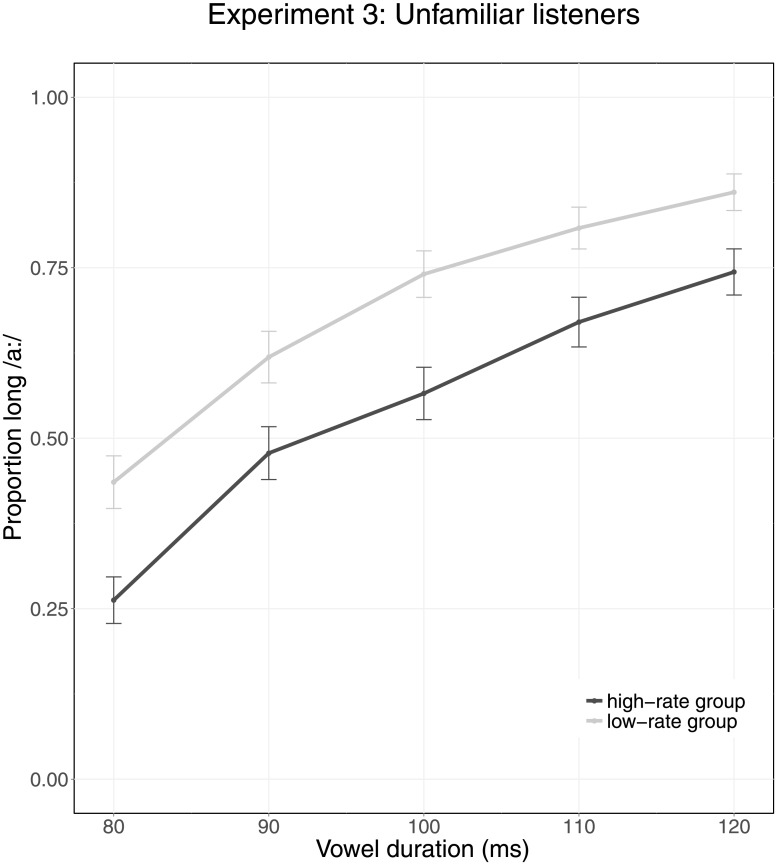
Average categorization data of Experiment 3 (unfamiliar listeners). The X-axis indicates Vowel Duration (80–120 ms). Colors indicate Group, with the high-rate group shown in dark grey and the low-rate group shown in light grey. Error bars represent the 95% confidence intervals.

Fig 5 presents the categorization data of Experiment 3. The figure shows that participants reported higher proportions of long /a:/ for target vowels with longer durations. The difference between the two lines suggests that participants in the high-rate group reported hearing fewer long vowels than the low-rate group.

The full GLMM as described in Experiment 1 tested the categorization data of Experiment 3. The model revealed a significant effect of Vowel Duration (*β* = 1.195, *z* = 5.298, *p* < 0.001), with the proportion of /a:/ responses increasing for longer vowel durations. Moreover, a significant effect of Group was observed between the high-rate group and the low-rate group (*β* = 1.064, *z* = 2.895, *p* = 0.004), with the high-rate group (who listened to fast and neutral speech) reporting a lower proportion of /a:/ than the low-rate group (who listened to slow and neutral speech). The model revealed no significant effects of Block (*β* = −0.163, *z* = −1.469, *p* = 0.142) and the control variable Talker (*β* = 0.196, *z* = 0.641, *p* = 0.522) on vowel categorization. None of the interactions between predictors reached significance (Groups and Vowel Duration: *β* = 0.135, *z* = 0.469, *p* = 0.639; Groups and Block: *β* = −0.173, *z* = −1.234, *p* = 0.217; Vowel Duration and Block: *β* = 0.049, *z* = 1.268, *p* = 0.205).

We performed an omnibus analysis on the combined data from all three experiments, to test whether the group effects in each experiment were significantly different from each other. A GLMM was run, comprising Group (sum-to-zero coded: slow coded as 0.5, fast as −0.5), Experiment (dummy coded, with Experiment 3 mapped onto the intercept), Vowel Duration, Block, and Talker, as well as the interaction between Group and Experiment. This model revealed two significant interactions. First, the interaction between Group and the contrast between Experiments 1 and 3 was significant (*β* = −1.262, *z* = −14.72, *p* < 0.001), demonstrating that the contrast between Groups in Experiment 1 was significantly different from the contrast in Experiment 3. Similarly, the interaction between Group and the contrast between Experiments 2 and 3 was significant (*β* = −1.014, *z* = −10.19, *p* < 0.001).

The results of Experiment 3 show that the global speech rate of an unfamiliar talker affects perception of temporally ambiguous vowels in another talker’s speech; neutral rate speech sounds slow in the presence of a faster talker and vice versa. The results replicate the finding in Maslowski et al. [31] that the global rate of one talker is perceived relative to the global rate of another talker. Furthermore, the results of Experiment 3 indicate that the results obtained in Experiment 1 and 2, in which no differences between groups were found, were due to recognition of one’s own voice. Finally, the results indicate that global rate effects are resilient to small variations in speech rate. In contrast to the (artificially compressed/expanded) fast and slow speech in Maslowski et al., the speech recorded in Experiment 1 was natural speech, exhibiting slight variability in speech rate both within and between sentences. Therefore, Experiment 3 demonstrates that listeners can rely on roughly stable global speech rates.

## Discussion

This study investigated the involvement of self-produced speech in perception of another talker’s global speech rate. In each of the three experiments, two groups of participants listened to and evaluated neutral speech rate trials from another talker. In Experiment 1, these perception trials were interspersed with production trials in which a high-rate group was instructed to produce speech at a (pre-specified) fast rate, whereas a low-rate group was instructed to produce speech at a slow speech rate. We measured the difference in perception of the Dutch /α, a:/ vowel contrast in the neutral rate speech from the other talker between the two groups. The results indicated that self-produced speech did not influence rate perception of the other talker (i.e., there was no difference between groups).

Because Experiment 1 could not exclude the possibility that speaking-induced suppression (SIS: reduced auditory response to self-produced speech) veiled a potential effect of self-produced speech, we performed another experiment to test this account. Experiment 2 was identical to Experiment 1, but this time, the participants from the first experiment listened to playback of their own speech, whilst evaluating target vowels in neutral rate speech as before. Again, no group difference was found on perception of neutral rate speech from another talker, indicating that the absence of a group effect in Experiment 1 was not a by-product of SIS.

Experiment 3 was conducted to confirm that the absence of global rate effects in the preceding two experiments was due to participants listening to themselves. In Experiment 3, a new participant sample performed the task of the second experiment. As such, the participants listened to two unfamiliar talkers, one of which was a prior participant. Here, the global speech rate effect previously observed in Maslowski et al. [31] was replicated; neutral rate sounded slow in the context of a faster talker (as evidenced by a lower proportion of long /a:/ responses), but fast in the context of a slower talker (higher proportion /a:/). Moreover, this global rate effect emerged in naturally produced speech contexts, showing for the first time that this effect is robust against small within-talker rate variability.

The results of the experiments provide valuable clues to which aspects of our own productions play a role in perception. In the literature, talkers have been suggested to be aware of sub-phonemic details in their own speech. For instance, talkers make online corrections when receiving altered auditory feedback during a speech production task [38, 39]. Moreover, perception can be facilitated when stimuli are self-produced in L1 speech [40], L2 speech [41, 42], and in action perception [7, 8]. Studies of the neurobiological correlates of speech perception have argued that processing advantages for self-produced speech are due to a self-awareness network involving mirror-like systems [43, 44]. Interestingly, self-benefit seems to occur only when listeners recognize their own voice [40, 45]. These findings suggest that listeners must be very sensitive to their own voice, which is processed differently from other talkers’ voices [46].

Our experiments support that one’s own voice must somehow be marked in comparison to other talkers’ voices. The lack of effects of self-produced speech in Experiment 1 and 2 may consequently be a result of participants strategically ignoring their own productions, regardless of whether they were listening to themselves whilst speaking or during passive listening. This may be due to reduced attention when listening to one’s own voice [47]. The findings are in line with a study on explicit judgments of speech rate by Koreman [48]. Koreman found no systematic differences in speech rate perception of others as a function of a listener’s own habitual articulation rate or (clear vs. sloppy) speaking style (but see [49]), suggesting that talkers disregard feedback from their own speech rate when listening to others.

Another possible interpretation of the absence of context effects in Experiments 1 and 2 is that the global self-produced speech rates produced by the participants were not their habitual rates. The participants listening to self-produced speech had an enormous amount of prior experience with their own habitual rates, and, consequently, the artificial and imposed rates at which they had to speak in the first experiment may have had little impact on the perception of another talker’s rate in both experiments.

Both interpretations of the results support the involvement of self-awareness in perception of self-produced speech. From the data presented here, we cannot distinguish specifically between awareness of hearing oneself and awareness of the self-produced speech rate in the lab not being representative of one’s habitual rate. Therefore, manipulating awareness could be an interesting avenue for future research to enhance our understanding of the involvement of self-awareness in perception of self-produced speech. However, given the other facets of cognition in which self representations seem to be different from representations of others, we argue that it is more plausible that the effects were due to self rather than to familiarity with one’s own habitual rate as a consequence of greater exposure.

Both of these accounts are consistent with episodic models of speech perception. In episodic models, word recognition is shaped by distributional properties coming from detailed representations (i.e., exemplars) of every instance of a word in the input [50, 51]. If our results stem from great prior exposure to one’s own production, the extensive experience listeners have with their own speech productions could have led to richer and more robust representations of their own voices [52]. This would restrict another talker’s speech to be perceived relative to one’s own new tokens produced at an extraordinary and artificially imposed speech rate. However, exemplars are also assumed to be labeled for various indexical features, such as talker voice [53]. If our findings are a result of self-produced speech being encoded as such (i.e., with a talker-specific label for ‘self’), one’s own voice may consequently be ignored in perception of others.

The lack of a context effect in Experiment 1 is particularly interesting in relation to findings by Bosker [22]. Bosker compared participants’ vowel categorization immediately after having produced either fast or slow speech. As soon as participants had produced a sentence, they would hear an ambiguous target word from another talker. In this very local self-produced speech context, Bosker observed a difference in participants’ perception of target words, suggesting that a talker’s own speech can modulate perception of another talker when immediately preceding an ambiguous word. Experiment 1 shows, however, that in larger contexts, incoming speech is not necessarily encoded with reference to representations reflective of listeners’ own productions.

Bosker [22] also found an enhanced effect of self-produced speech when participants listened to playback of their own speech relative to a production experiment, which he speculated may have been a consequence of SIS in the production experiment. The difference between the local rate-dependent effects in Bosker [22] and the null effect in our Experiment 2 suggests that local and global speech rate normalization involve different mechanisms.

Local and global rate-dependent context effects may be interpreted with reference to Bosker et al.’s [28] two-stage model of normalization processes in speech perception. This two-stage model includes a first stage that is related to early perceptual adjustments, involving online low-level processing of temporal and spectral information in the signal. This first stage includes effects of local surrounding contexts, which are obligatory [54], happen prelexically, are independent of talker changes [22, 23], not specific to speech contexts [11, 16, 24, 25, 55], and continue to exist under cognitive load [28]. Because perceptual normalization is automatic, self-produced speech rate—either actively produced or passively heard [22]—in *local* contexts directly modulates perception of others.

The second stage involves domain-specific cognitive adjustments performed later in time (rather than perceptual normalization), after talker segregation. Here, word recognition may be modulated by comparing the speech input to an expected form considering a certain speech context or talker [19, 56, 57]. Therefore, talker-specific global speech rate effects, such as the effect reported in Maslowski et al. [31], likely occur during the second stage. Importantly, feedback from one’s own present speech rate is disregarded in global speech rate normalization; regardless of whether listeners hear themselves actively or passively, they ignore their own speech in perception of another talker. Whether talkers’ own habitual rates play a part in the online processing of others’ speech remains to be determined. Yet, such an influence is argued to be unlikely, since adjustments based on one’s own speech would not facilitate perception of other talkers. That is, tracking self-produced speech rate presumably does not facilitate comprehension of others’ speech, whereas tracking other talkers’ speech rates may help perception in the long term.

## Conclusion

The current study shows that listening to one’s own voice is special: Self-produced speech is processed differently from speech produced by others (Experiment 1). This seems to be task independent, as playback of one’s own speech also does not elicit an effect of self-produced rate on perception of others (Experiment 2). Furthermore, this study shows that global rate effects can be replicated with naturally produced speech (Experiment 3). Importantly, this indicates that some amount of within-talker variability in speech rate is allowed before global rate tracking fails. These findings shed further light on the complex mechanisms of speech perception in dialogue settings, highlighting the hierarchical processes involved in rate normalization, as suggested by the two-stage model in Bosker et al. [22]. To further empirically test this model, future work may investigate the time course of global rate effects to explore timing differences between local and global rate normalization.
